# Cancer immunotherapy: avoiding the road to perdition

**DOI:** 10.1186/1479-5876-2-26

**Published:** 2004-07-29

**Authors:** Maurizio Chiriva-Internati, Fabio Grizzi, Robert K Bright, W Martin Kast

**Affiliations:** 1 Department of Microbiology & Immunology and Southwest Cancer Treatment and Research Center, Texas Tech University Health Science Center, Lubbock, TX, 79430, USA; 2 Scientific Direction, Istituto Clinico Humanitas, Rozzano, 20089 Milan, Italy; 3 "M. Rodriguez" Foundation – Institute for Quantitative Methods in Medicine, 20100 Milan, Italy; 4 Norris Comprehensive Cancer Center, University of Southern California, Los Angeles, CA, 90089 USA

## Abstract

The hypothesis that human cancers express antigens that can be specifically targeted by cell mediated immunity has become a scientifically justifiable rationale for the design and clinical testing of novel tumor-associated antigens (TAA). Although a number of TAA have been recognized and it has been suggested that they could be useful in the immunological treatment of cancer, the complexity of human beings leads us to reflect on the need to establish new criteria for validating their real applicability. Herein, we show a system level-based approach that includes morphological and molecular techniques, which is specifically required to improve the capacity to produce desired results and to allow cancer immunotherapy to re-emerge from the mist in which it is currently shrouded.

## Introduction

Although considerable advances have been made in terms of our molecular and cellular knowledge, for most human disease states a fundamental understanding of causal disease onset, disease mechanism and progression, and optimal treatment is still significantly limited.

In part, this advancement has been hampered by our inability to fully and rapidly delineate complex cellular metabolic processes and molecular pathways.

Organisms are complex self-organizing entities made up of such parts: organs, tissues, cells, organelles and ultimately molecules and atoms. One question that arises, concerns the relationship between the whole and its component parts. The issue at stake is sometimes called "the question of reduction" or "the problem of reductionism" [1].

The inefficacy of contemporary science to describe biological systems, consisting of non-identical parts that have different and non-local interactions has tended to limit progress in the human healthcare. Many biological systems remain incomprehensible because their multifarious nature has been combined with a reductionist approach based on the linear conception of *cause *and *effect*.

The use, however, of a more *holistic *multidimensional system level-based approach may provide new insights into the understanding of disease processes and mechanisms of action of therapeutical agents [2].

Herein we aim to introduce a system level-based approach that includes morphological and molecular techniques for validating the appropriateness of using novel tumor-associated antigens (TAA) for clinical purposes. This approach might be easily implemented for identifying prognostic, diagnostic and alternative biomarkers.

Finally, this type of analysis of appropriately designed cohorts might also provide a key to understanding the differences in patients who do or do not respond to any particular therapy. This information may be helpful for a more effective (and therefore more *cost-effective*) design of clinical trials [2].

## Immunotherapy and the human complexity

The recognition and characterization of novel TAA is fundamental to the advance of cancer immunotherapy. The original hypothesis of Boon [3] and Rosenberg [4] that human cancers express antigens that can be specifically targeted by cell mediated immunity has become a scientifically justifiable rationale for the design and clinical testing of novel TAA based immunotherapies and therapeutic vaccines [5-7].

However, although a number of TAA have been discovered and it has been suggested that they could be useful in the immunological treatment of cancer, the *complexity *of human beings leads us to reflect on the need to establish compelling new criteria for validating their real applicability. *Biological complexity *can be intuitively appreciated – at least in terms of morphological or behavioral complexity, or the variety of cell types in an organism – but the term itself is notoriously difficult to define [8]. Human beings are *complex hierarchical system*s consisting of a number of *levels of anatomical organization *(genes, cells, tissues, organs, apparatuses, and organism) that interrelate differently with each other to form networks of growing complexity. The concept of anatomical entities as hierarchy of graduated forms, and the increasing number of known structural variables, have highlighted new properties of organized biological matter and raised a series of intriguing questions. In order to understand biology at the system *level*, we need to examine the structure and dynamics of the functions of organisms rather than the characteristics of their constitutive isolated parts [8-13].

The expression of TAA in biological materials has mainly been studied at the level of gene expression and gene level measurement by Reverse Transcriptase-Polymerase Chain Reaction (RT-PCR) analysis and the Quantitative real-time PCR (qrt-PCR) technology [14-17]. However, the *information *provided by these approaches is limited by the fact that the phenomena observed at each level of anatomical organization have properties that do not exist at a lower or higher level: RT-PCR and qrt-PCR may offer a satisfactory *qualitative/quantitative *description of *small-scale structures*, but this is likely to be irrelevant when it comes to *large-scale features*. The above considerations, in conjunction with the *complexity of tumor-host interactions *within the tumor microenvironment caused by temporal changes in tumor phenotypes and an array of immune mediators expressed in the tumor microenvironment [18] might clarify the limited reliability and applicability of current immunotherapeutic approaches.

**Figure 1:**
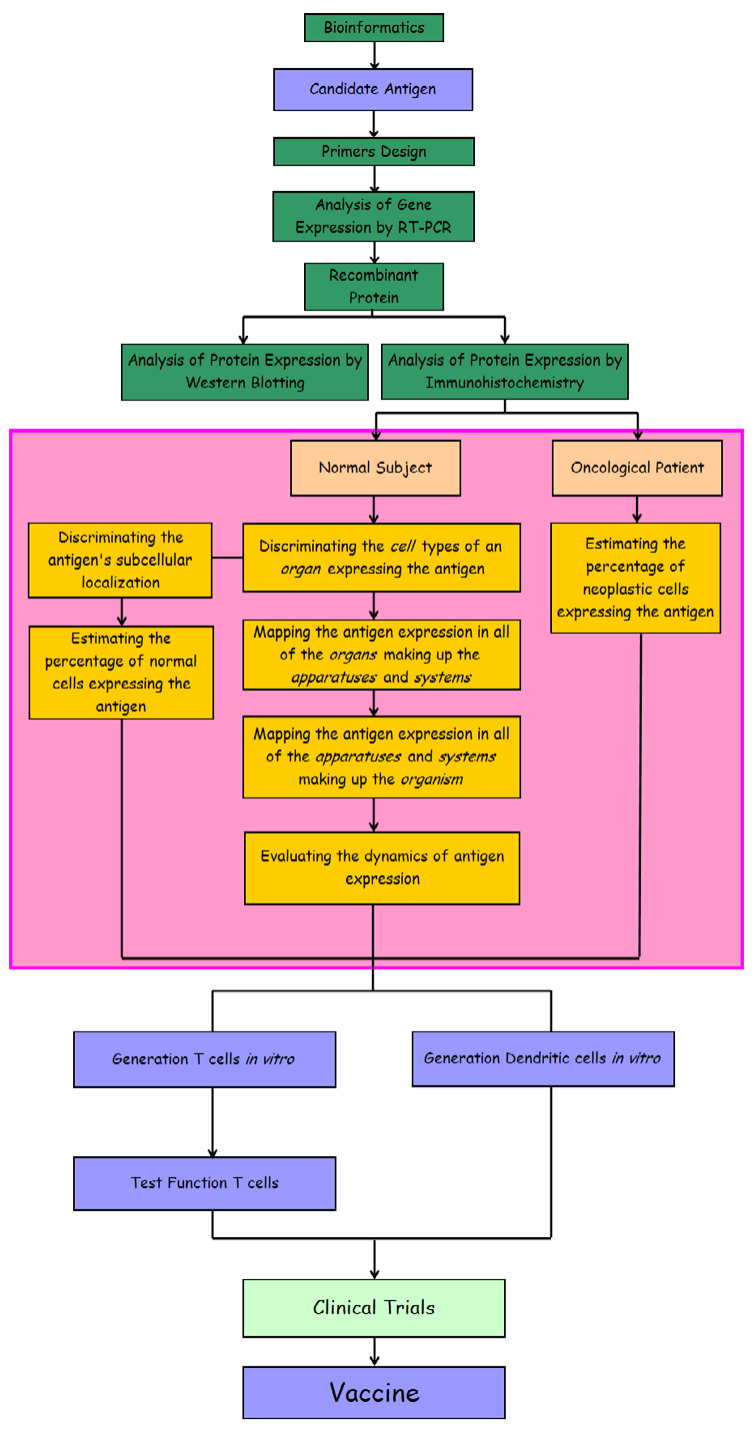
System level-based approach for validating candidate TAA for clinical application. Aside from the well defined experimental procedure, the method presented here is based on the *complex hierarchical nature *of the human beings. The analysis begins at level of gene expression and then continues to higher levels of anatomical organization, (cell, tissue, organ, apparatuses and organism). This approach includes both morphological and molecular techniques. It also introduces the concept of dynamics of TAA expression at the level of the *cell cycle*, the *physiological status of the organism *and the *process of aging*.

Here, we suggest a *system level-based *approach (Figure 1) for validating the appropriateness of using TAA for clinical purposes, which includes the following never defined before key points:

• *Discriminating *the *cell types *expressing the candidate antigen on the basis of the morphological visualization of all of the parts making up the *organ *under investigation.

• *Discriminating *the candidate antigen's sub-cellular localization (at the level of cell *nucleus*, *cytoplasm *and/or *plasma membrane*) by ultra-structural morphological visualizations.

• *Mapping *candidate antigen expression in all of the *organs *making up the *apparatuses*.

• *Mapping *candidate antigen expression in all of the *apparatuses *making up the living *organism*.

• *Estimating *the percentage of normal cells and their neoplastic counterparts expressing the candidate antigen.

• *Evaluating *the *dynamics *of candidate antigen expression at the level of the *cell cycle*, the *physiological status of the organism *(*i.e. *the woman's menstrual cycle) and the *process of aging*.

In order to advance our knowledge in a currently widely debated field of investigation, a clearer distinction must be made between *in vitro *laboratory results (the discovery and validation of target antigens) and their *in vivo *application (*in vivo *validation), and it is necessary to adopt a more complete experimental approach that forcefully includes both morphological and molecular techniques [19].

## Conclusions

Translational science which is aimed to test, in humans, novel therapeutic strategies developed through experimentation [20] should begin to consider the role of *emergence *in other words the appearance of *unexpected structures *and/or the occurrence of *surprising behaviors *in large systems composed from microscopic parts, whether physical or biological. By unexpected and surprising we mean structures and behaviors which are not *intuitive *and are not simply *predictable*.

Since our understanding of complex human disease such as cancer, is still limited and pre-clinical models have shown a discouraging propensity [2,6] to fail when applied to humans, a new way of thinking is strongly needed that unites physicians, biologists, mathematicians and epidemiologists, in order to develop a better theoretical framework of tumor development, progression and tumor-host interactions.

Although the model presented here is based on a multidisciplinary system-level approach probably within the reach of only very large and multi-talented laboratories, it is aimed to introduce a different way of investigating *human cancer, *which takes into account the complexity of the human being as a system.

The use of a holistic approach, which enables a more accurate selection of immunotherapeutic target antigens in the first phase of the experimental research, will reduce the notable fragmentation of the biological information in the post-genomic era, and will facilitate a more accurate transfer of the acquired knowledge to the bedside.

Further, this new multidisciplinary approach is specifically required to improve the capacity to produce desired results with a minimum expenditure of *energy*, *time*, or *resources *for immunotherapeutic treatments and to allow cancer immunotherapy to re-emerge from the mist in which it is currently shrouded.
